# Quantitative evaluation of robust skull stripping and tumor detection applied to axial MR images

**DOI:** 10.1007/s40708-016-0033-7

**Published:** 2016-02-01

**Authors:** Ahmad Chaddad, Camel Tanougast

**Affiliations:** ASEC Team at the LCOMS Laboratory, The University of Lorraine, Metz, France

**Keywords:** Image segmentation, MRI brain, Similarity measure, Skull stripping, Tumor

## Abstract

To isolate the brain from non-brain tissues using a fully automatic method may be affected by the presence of radio frequency non-homogeneity of MR images (MRI), regional anatomy, MR sequences, and the subjects of the study. In order to automate the brain tumor (Glioblastoma) detection, we proposed a novel approach of skull stripping for axial slices derived from MRI. Then, the brain tumor was detected using multi-level threshold segmentation based on histogram analysis. Skull-stripping method, was applied by adaptive morphological operations approach. This is considered an empirical threshold by calculation of the area of brain tissue, iteratively. It was employed on the registration of non-contrast T1-weighted (T1-WI) and its corresponding fluid attenuated inversion recovery sequence. Then, we used multi-thresholding segmentation (MTS) method which is proposed by Otsu. We calculated the performance metrics based on the similarity coefficients for patients (*n* = 120) with tumor. The adaptive algorithm of skull stripping and MTS of segmented tumors were achieved efficient in preliminary results with 92 and 80 % of Dice similarity coefficient and 0.3 and 25.8 % of false negative rate, respectively. The adaptive skull stripping algorithm provides robust skull-stripping results, and the tumor area for medical diagnosis was determined by MTS.

## Introduction

Brain images provide signals of brain anatomy and can be useful in diagnosis of numerous brain abnormalities such as malignant glioma [1]. Tumor and skull have resembled intensity which makes automatic tumor detection difficult. To overcome this challenge, skull-stripping algorithm is desired as a pre-processing step for detecting the brain tumor.

**Fig. 1:**
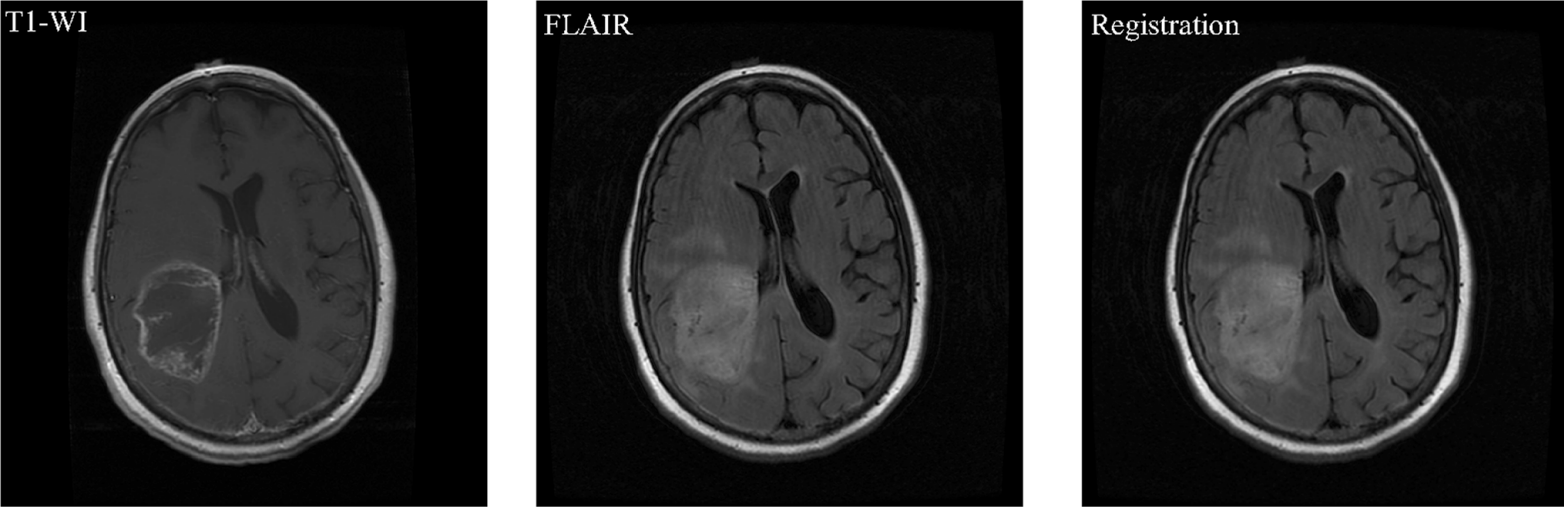
Example of axial brain image with resembles intensity value of both skull and tumor. Brain image within tumor on T1-WI, the corresponding axial FLAIR sequence, and the corresponding rigid registration based on fixed image T1-WI and moving image FLAIR sequence

Similar tumors have different imaging features on T1-WI, when compared to T2 weighted and FLAIR. Numerous malignant brain tumors can be seen by registration technique which is a process of aligning images from different modalities using the translating, rotation, and various scales [2, 3]. This registration can be done by fixing T1-WI image and moving its FLAIR corresponding image (Fig. 1). After this step, skull stripping is preferred to apply, and then whole abnormalities area can be detected by MTS.

For skull-stripping algorithms, three approaches, namely, region-based, edge-based, and hybrid methods are used currently which are also being used for segmentation in numerous domains.

Using region-based methods, the brain tissue can be distinguished from surrounding tissues like fat, bone, and muscles. Brain regions can be extracted by morphology operators such us erosion, dilation, opening, and closing using many techniques such as region growing and watershed [4–8]. Fully automated 3D skull-stripping algorithm was done independent of scan orientation [4]. Histogram analysis and morphological operation have been considered for achievement of skull stripping [4, 7]. Morphological operations are simple and fast methods, and can be applied with statistical techniques for 3D skull stripping [5]. Another algorithm of skull stripping works based on foreground/background thresholding, and isolates the brain, skull, and head tissues using the morphological operations [8]. Morphological operation-based method is relativity sensitive to isolate enough brain tissues, similar to brain extraction tool (BET), [9, 10]. Watershed techniques have been employed but it is sensitive to the noise factors [11]. The other techniques are dependent on predefine criteria, such as the growing region which is based on pixels groups, and cannot be fully automated because they need a user to set the prior information [12].

Edge-based methods as a level set and snake algorithms which are based on minimizing total image energy for detecting brain tissue. In this term, model-based level set algorithm has been applied for robust skull stripping [10]. However, these techniques based on edge which is sensitive by noise factors, need the contour initialization by user and have a high time computation.

Hybrid methods included combined region and edge techniques. For example, combined anisotropic diffusion filtering, edge detector, and morphology operations have been applied to enhance the automate process, such as brain surface extractor (BSE), [13]. Combined multiple results of various skull-stripping techniques have been analyzed and discussed for showing the advantage and disadvantage for each proposed method [14, 15]. Additionally, numerous algorithms have been employed and showed limited success in large-scale data [16, 17]. Also, low contrast levels and connections between the brain and surrounding tissues can be a problem for these algorithms. It can be a difficult task for automating skull stripping without initialization of parameters and high execution time. Fully automated skull-stripping methods should have the capability to extract the brain accurately from a large database of T1-WI MRI of head scans without any user intervention according to Somasundaram et al. [18].

Since sometimes there are not enough boundaries between brain and the bone, intensity of the skull can be read as tumor enhancing portion in automated segmentation. This paper is presenting a specific skull-stripping method applying when there is not enough space between brain tissue and skull.

In order to overcome the limitations of skull stripping and segmentation methods and in order to obtain more automate processing, we propose a fully automated tumor detection by registered T1-WI and its corresponding FLAIR sequence, skull stripping using the iterative morphological operations, and MTS to detect the brain tumor.

The rest of this paper is organized as follows: The methods section describes the implementation of the proposed algorithm; the results section summarizes the results and performance assessment metrics. The discussion section discusses the proposed iterative morphological method and tumor segmented based on MTS method of this research to solve robust of the skull-stripping and tumor-detection problem.

## Materials and methods

### Patient and data acquisition

**Fig. 2:**
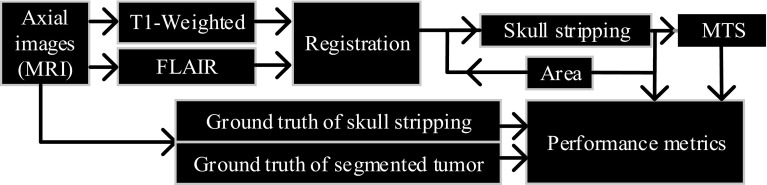
Schematic diagram of the proposed method for adaptive skull stripping and tumor detection based on MTS. Skull-stripping algorithm applied using the registration output, and MTS works based on the output from skull-stripping algorithm

In this study, data from 120 glioblastoma multiforme (GBM) patients (age: average = 57, median 56, minimum = 34, and maximum = 81) were collected from the cancer imaging archive (TCIA) database (http://cancerimagingarchive.net/) and used to validate the proposed method. The GBM data were acquired prior to any treatment from patients with brain tumors that were subsequently diagnosed as GBM. 3D Slicer software was used for illustrating the GBM tumor and testing that the patient images can be correctly registered using non-contrast T1-WI and FLAIR sequence. Schematic represents the proposed method for the skull stripping and tumor detection using raw data from MR images (Fig. 2). The images are converted into grayscale before further processing. A description provided on pre-processing, followed by the adaptive skull stripping and MTS for tumor detection. Only T1-WI and FLAIR sequences were used for evaluating the proposed algorithm of skull stripping and MTS. All of the images had 512 × 512 pixels acquisition matrices, and only one slide image including the GBM tumor with its phenotypes (Necrosis, active tumor/contrast enhancement and edema/invasion) from each patient was collected for skull tripping and segmentation process. The imaging protocol used whole-brain T1-WI and FLAIR scanning using a 3T MRI scanner (GE-Healthcare). T1-WI and FLAIR scans were acquired based on the slice thickness (ST) = 5 mm.

### T1-WI- and FLAIR-based registration

**Fig. 3:**
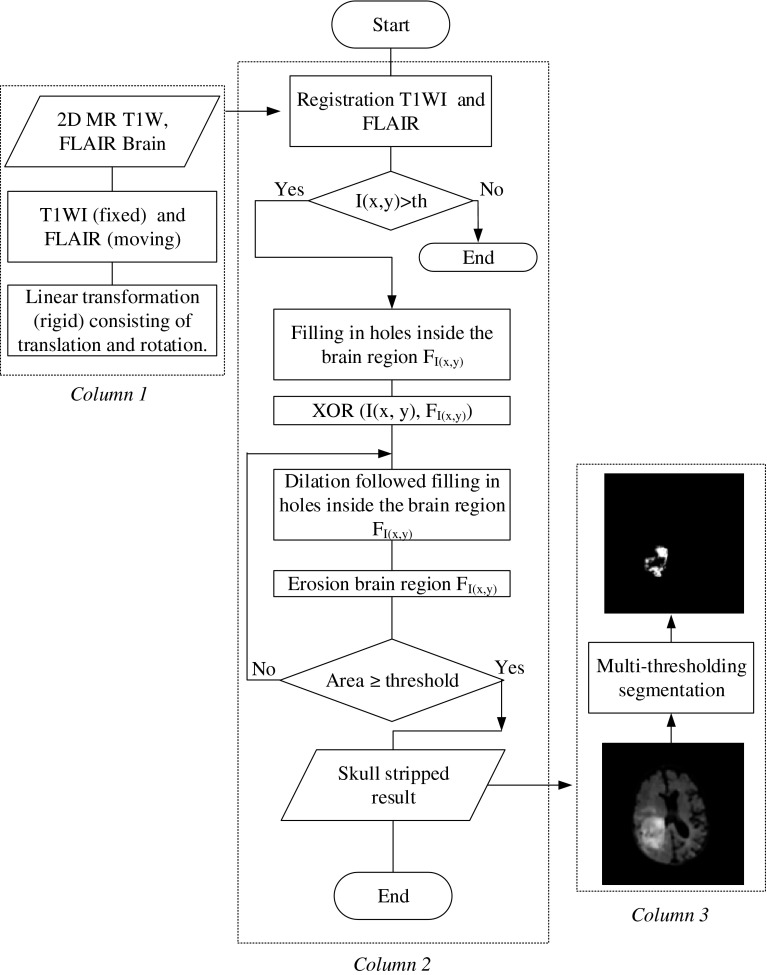
Flowchart of the skull-stripping and tumor-detection algorithm. Registration, iterative design within morphological operators of the proposed skull-stripping algorithm and MTS algorithm

Registration is based on transformation by using several factors such as translating and rotating [2, 3]. Many algorithms have been proposed, however, two principal registration approaches used mostly are as follows: feature- and intensity-based registration. Feature-based registration (FBR) works for the identification of corresponding points in the two images namely, fixed image (T1-WI) and a moving image (FLAIR). In volume data, multiple of landmark methods is used to establish a rigid transformation between two volumes [19]. Note that the errors decreased when the number of points increased [20, 21]. In this study, intensity-based registration (IBR)/voxel similarity-based is considered. It works by applying a transformation to the source image which computed a value for how similar it is to the target. More precisely, it works based on the iterative process, and the source will generally be transformed many times until the two images performed and achieved highest similarity [2, 22, 23]. We aligned and registered the scans to each other. Moreover, most of the voxel size of the FLAIR, and T1-W1 images were similar and simply registered. However, in case that the voxel size was dissimilar, we resampled the FLAIR volume to the matrix of T1-W1 voxel size. The patient’s images which have complex rotation modifications and registrations were not considered in order to achieve an error <2 mm. The average of computational time necessary to complete each volume registration is 40–50 s, (Fig. 3, column 1). An example shows the registration based on the corresponding T1-WI and FLAIR sequences (Fig. 1). And registration for each patient’s data was done by using T1-WI and its corresponding FLAIR sequence using Matlab software.

### Proposed skull-stripping algorithm

Skull stripping is performed based on morphological image processing (MIP) which is operated by passing a structuring element (SE) over the image in an activity similar to convolution [19]. Note that the SE can have different sizes and shapes, and is a sub-image. At each pixel position, a specified operation is applied between the SE and the matrix data of image. The created effect depends on the size, shape, content of the element structure, and the nature of the operation. Moreover, the choice of the SE is depending on the desired object within an image. A review of various fundamental MIP techniques, namely, erosion, dilation, opening, and closing is presented below. Binary and gray scale images have been considered in the morphological operations of skull stripping. Let *B* be a binary image and *S* be the SE containing any complement of ‘0’ and ‘1.’ Both defined on a 2D Cartesian grid. Denote by *S*_*xy*_ the structuring element after it has been translated so that its origin is located at the point (*x*, *y*). We employed the skull striping based on the following fundamental MIP techniques.

#### Erosion

Erosion is the process of shrinking an object in the image, leaving it smaller in area. The erosion of *B* by *S* is defined according to

1 $$B\,{ \ominus }\,S = \left\{ {(x, y)\left| {S_{xy} \subseteq B} \right.} \right\}.$$

Output result from *B* ⊖ *S* is a binary image from eroding *B* by *S*.

#### Dilation

It can expand an object in the image, leaving it larger in area. The dilation of *B* by *S* is defined as

2 $$B \oplus S = \left\{ {\left( {x,y} \right)\left| {\left( {S_{xy} \;{ \cap }\;B} \right) \ne \emptyset } \right|} \right\}.$$

The binary image *B* ⊕ *S* which is a result of dilating *B* by *S*, *S* is translated so that its origin is located at (*x, y*), and then its intersection with *B* is not empty.

Additionally, dilation followed by erosion is called closing. It uses for filling small and thin holes in objects, connecting nearby objects, and generally smoothing the boundaries of larger objects without significantly changing their area. It can be expressed according to

3 $$B \, \blacksquare \, S = \left( {B \oplus S} \right)\,{ \ominus }\,S.$$

For eliminating small and thin objects, breaking objects at these points, and generally smoothing the boundaries of larger objects without significantly changing their area. We use the opening process which is based on the erosion followed by dilation. It can be expressed according to

4 $$B \,{\circ }\,S = \left( {B\,{ \ominus }\,S} \right) \oplus S.$$

To perform the skull stripping, we used *XOR* function which is a logical exclusive-OR. It can be expressed between two images *B*(*x,y*) and *I*(*x,y*), according to

5 $$B\,XOR\begin{array}{*{20}c} {~I} \\ \end{array} = \left\{ {\begin{array}{*{20}c} {0 \quad {\text{if}}~\;B\left( {x_{i} ,~y_{j} } \right) = I\left( {x_{i} ,~y_{j} } \right)} \\ {1 \quad {\text{if}}\;~B\left( {x_{i} ,~y_{j} } \right) \ne I\left( {x_{i} ,~y_{j} } \right)} \\ \end{array} } \right.$$

where *i* and *j* are the indexes of pixel coordinates.

Figure 3 shows the flowchart algorithm of skull stripping by using the morphological operations. This algorithm is simply applied on 2D axial brain image for skull removing. The novelty of the algorithm is the threshold based on the iterative calculation of the brain material area. It is an adaptive algorithm that can be run and iterative numerous times to obtain the brain material images without skull based on two steps. It starts by using the IBR registration, then the output of IBR segments based on Otsu method which chooses a threshold to minimize the intra-class variance of the black and white pixels. Followed by the filling of all the holes in brain image *F*_*I*(*x,y*)_.
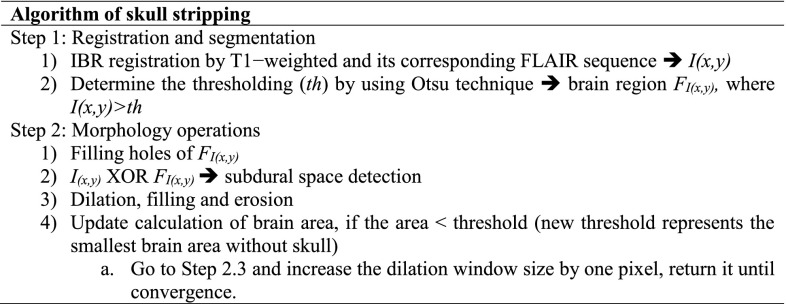


In this step, output is the binary image *I*_(*x,y*)_. Then, we obtained an image with a skull and brain area which is represented by the binary pixels ‘1.’ Using the XOR operator between *I*_(*x,y*)_ and *F*_*I*(*x,y*)_, we obtained the subdural space which is surrounding the brain as a contour, and by the dilation, filling, and erosion we can extract the brain without skull. Unfortunately, some images do not have enough subdural space, or have a thin space discontinuity which provides a problem for skull stripping. To resolve the problem, we increased the dilation window size by one pixel in each step of the loop, then the same window size of the filling and erosion similar area of original brain image was kept. Here, the threshold is an area (area: represents the number of pixels) of brain which was extracted in each step when the extracted brain area is greater than the threshold (th), it means that we obtained the brain without skull (status of convergence). Threshold was chosen by the empirical test of brain area computation in order to robust this process with minimum error.

### MTS-based tumor detection

We employed histogram thresholding-based segmentation on the skull stripped result for segmenting the brain tumor. Figure 3 shows an example of the tumor segmented based on MTS technique. To perform MTS, we applied Otsu’s method [24]), which is described below:

Consider the histogram of a magnitude image as a discrete probability density function (pdf), *p*(*i*):

6 $$p_{i} = \frac{{f_{i} }}{N}\quad{\text{and}}\quad\mathop \sum \limits_{i = 0}^{J} p_{i} = 1,$$

where *p*(*i*) ≥ 0, *f*_*i*_ is the frequency of the intensity level *I* and *N* is the total number of pixels in the image. Each pixel in the image assumes an intensity level from the set (*0, 1,* …*, J* − *1*), where *J* denotes the number of intensity levels or histogram bins.

The Otsu’s method assumes that the threshold image contains two classes of pixels or a bi-modal histogram with regions *r*_1_ and *r*_2_. It calculates the optimum threshold (*T*) separating those two classes so that their combined interclass variance is minimal. That is,

7 $$T = {\text{argmax}} \left\{ {p_{{r_{1} }} \left( d \right) \left[ {m_{{r_{1} }} \left( d \right) - m_{I} } \right]^{2} \,+ \,p_{{r_{2} }} \left( d \right) \left[ {m_{{r_{2} }} \left( d \right) - m_{I} } \right]^{2} } \right\},$$

where *m*_*I*_ is the mean intensity. By dividing the histogram into regions with intensity level *d*, the respective region probabilities can be expressed according to

8 $$p_{{r_{1} }} \left( d \right) = \mathop \sum \limits_{i = 0}^{d} p(i)$$

9 $$p_{{r_{2} }} \left( d \right) = \mathop \sum \limits_{i = d + 1}^{J - 1} p(i).$$

Note that the means of the respective regions are given according to

10 $$m_{{r_{1} }} = \mathop \sum \limits_{i = 0}^{d} \frac{{i\,{\cdot}\,p(i)}}{{p_{{r_{1} }} (d)}}$$

11 $$m_{{r_{2} }} = \mathop \sum \limits_{i = d + 1}^{dJ - 1} \frac{{i\,{\cdot}\,p(i)}}{{p_{{r_{2} }} (d)}}.$$

Moreover, all values of *d* are considered and the corresponding equations of *T* are evaluated. The intensity value, *d*, that produces the maximum sum of the class variance is chosen as the threshold value *T*. Similarly, it can extend to MTS of an image in order to find and detect the object (brain tumor) which is localized in one of these levels of image intensity. In order to automate brain tumor detection, we have to resolve the resemble noise pixels problem. This latter can be easily removed by the average filter size of 3 × 3 which is not affected the impact of the tumor detection.

### Performance metrics of skull stripping and GBM detection

Skull stripping was performed by using the implemented algorithm on MATLAB software. Also, tumors have been segmented automatically by MTS method. Ground truth of the whole axial images which was used in the process of skull stripping and tumor detection was prepared and reviewed by experts (three radiologists were manually performed the skull stripping and tumor segmentation by 3D Slicer) in order to evaluate the algorithms performance. We calculated four performance metrics namely, Jaccard similarity coefficient (JSC), Dice similarity coefficient (DSC), false positive rate (FPR), and false negative rate (FNR). Moreover, JSC and DSC measure the degree of correspondence between ground truth images and skull-stripping images [25, 26]. Similarly, we calculated the similarity metrics between the ground truth of tumor and segmented tumor of MTS method. JSC can be formulated according to

12 $${\text{JSC}}\left( {A,B} \right) = {{\left( {A \cap B} \right)} \mathord{\left/ {\vphantom {{\left( {A \cap B} \right)} {\left( {A \cup B} \right)}}} \right. \kern-0pt} {\left( {A \cup B} \right)}},$$

where *A* is the area of the brain region in the ground truth skull-stripped image and *B* is the area of the brain region of the corresponding image with skull stripped using the proposed algorithm. Additionally, the JSC of 1 represents complete overlap. Whereas an index of 0 represents that there are no overlapping pixels.

DSC was also employed to describe the overall level of similarity between automatic and ground truth of skull stripping. In this term, DSC has been calculated according to the following equation

13 $${\text{DSC}}\left( {A,B} \right) = {{2\left( {A \cap B} \right)} \mathord{\left/ {\vphantom {{2\left( {A \cap B} \right)} {\left| A \right| \cup \left| B \right|}}} \right. \kern-0pt} {\left| A \right| \cup \left| B \right|}}.$$

Moreover, false positive rate (FPR) and the false negative rate (FNR) were used to quantify over and under segmentation. Both FPR and FNR were calculated according to

14 $${\text{FPR}}\left( {A,B} \right) = {{\left( {A/B} \right)} \mathord{\left/ {\vphantom {{\left( {A/B} \right)} {\left( {A \cup B} \right)}}} \right. \kern-0pt} {\left( {A \cup B} \right)}}$$

15 $${\text{FNR}}\left( {A,B} \right) = {{\left( {B/A} \right)} \mathord{\left/ {\vphantom {{\left( {B/A} \right)} {\left( {A \cup B} \right)}}} \right. \kern-0pt} {\left( {A \cup B} \right)}}.$$

We can find a direct relation between JSC, FPR, and FNR according to the following expression:

16 $${\text{JSC}}\left( {A,B} \right) = 1 - {\text{FPR}} - {\text{FNR}}.$$

## Experimental results

Skull stripping is a challenging and critical component of image processing and in particular the MRI images post-processing. Automate processing has a variety of problems which require a pre-processing manual intervention to be resolved. We employed and validated the proposed approaches by the comparative study using ground truth and skull stripping and segmentation results from the automatic algorithm. All algorithms were simulated using MATLAB R 2013a (Mathworks Inc., Novi, MI, USA). We considered 120 patients with the brain tumor for assessing the proposed algorithms.

**Fig. 4:**
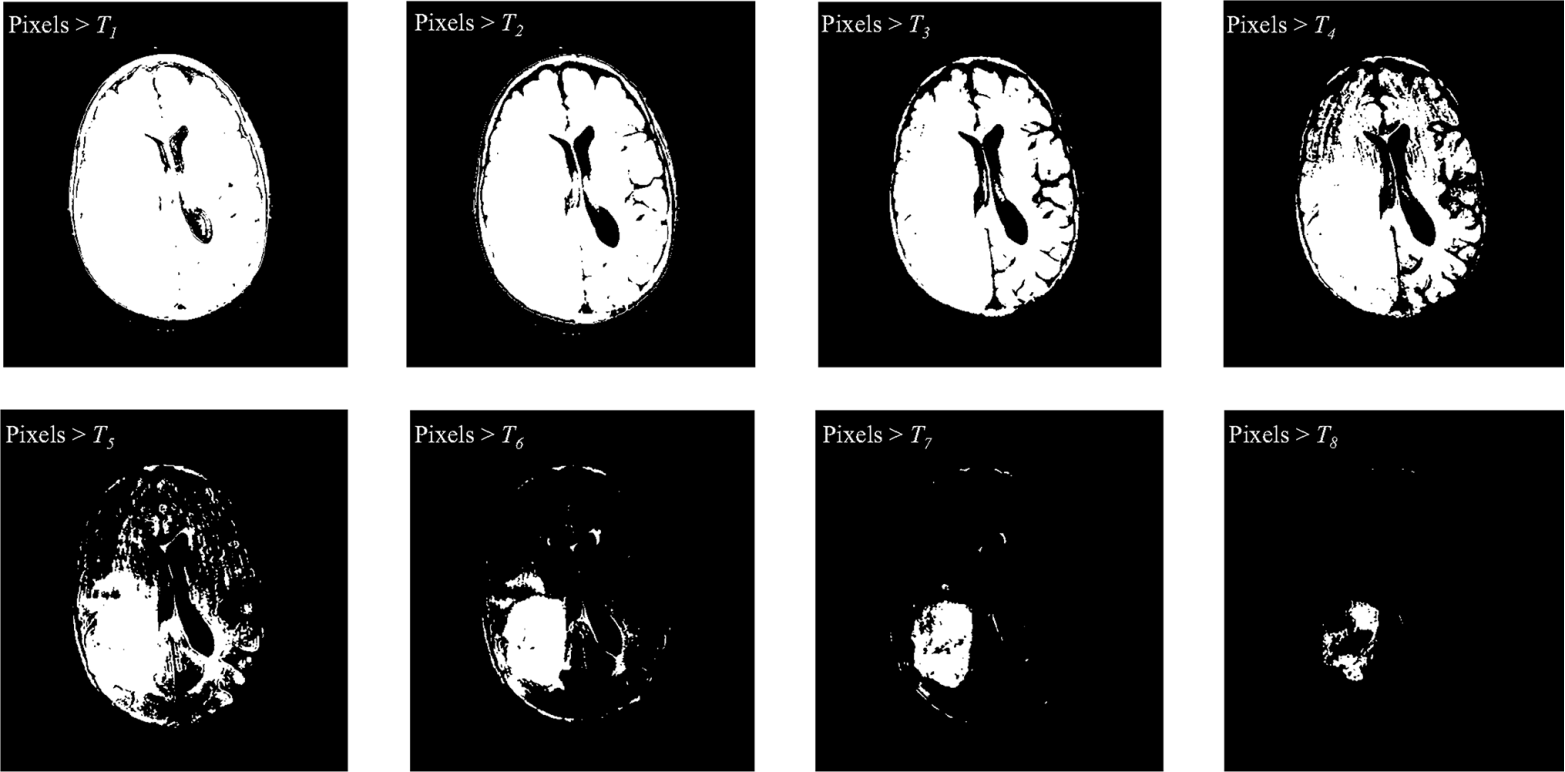
Tumor detection by using MTS. Eight thresholds provided based on Otsu’s method, where *T*_1_ = 0.03, *T*_2_ = 0.11, *T*_3_ = 0.18, *T*_4_ = 0.22, *T*_5_ = 0.26, *T*_6_ = 0.29, *T*_7_ = 0.35, *T*_8_ = 0.4

Figure 4 shows the flexibility of MTS based on Otsu’s method. It can provide several thresholds based on the previous equations [7–11]. Tumors can be detected by using the optimum threshold *T*_8_. The output result based on *T*_8_, is the tumor with some noises which can be easily removed using the average or median filter. Note that the input images are the output of skull stripped (see Fig. 3, column 2 and 3).

**Fig. 5:**
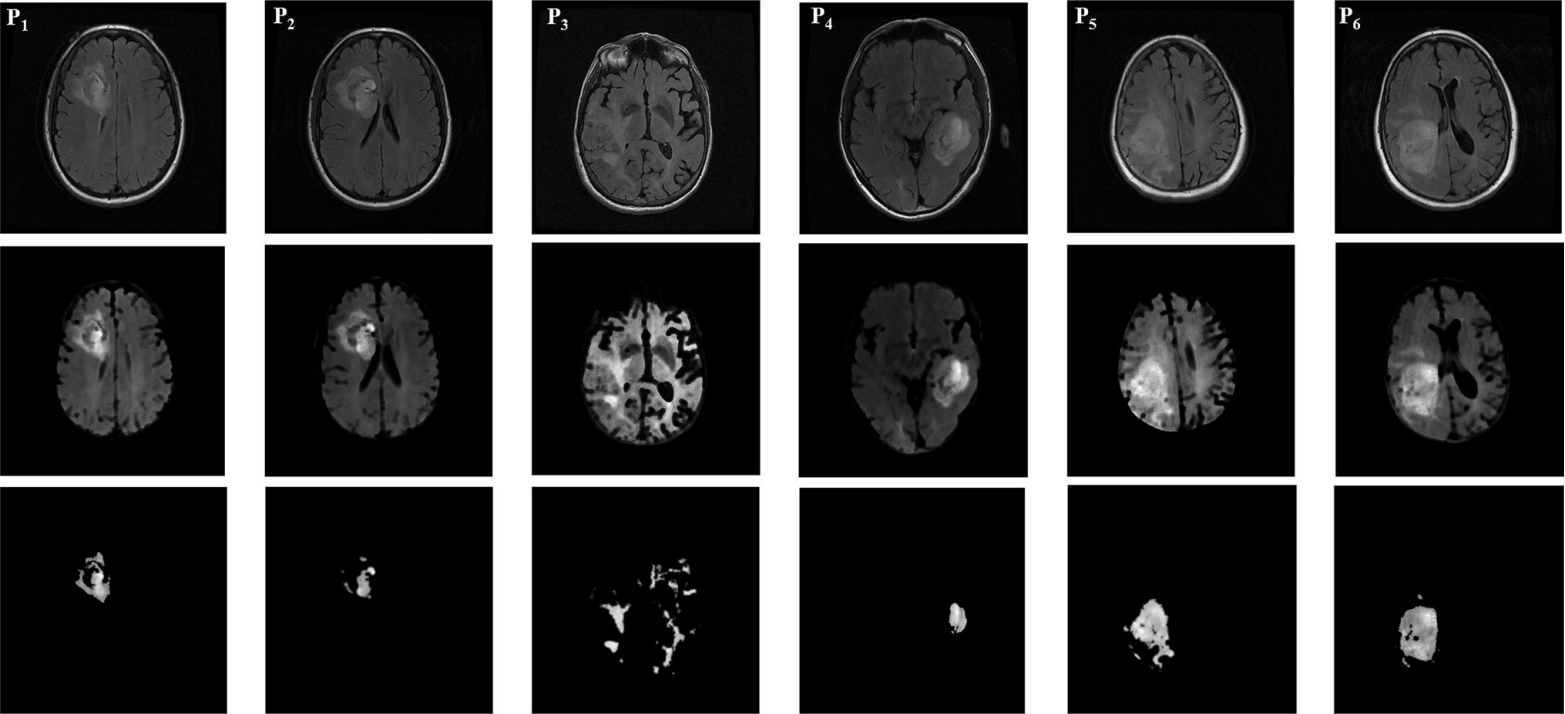
Registration, skull stripped, and tumor segmentation result of the proposed approach on 2D axial MR images. Example of six cases of GBM patients, namely *P*_1_, *P*_2_, *P*_3_, *P*_4_, *P*_5_, and *P*_6_, registration based on fixed image T1-WI and its corresponding mobile image FLAIR sequence (*first row*). Skull stripped applied on the output registered using the proposed algorithm (*second row*) and tumor segmented by using multi-thresholding segmentation based on Otsu’s approach (*third row*)

Figure 5 shows an example of six cases with registration, skull stripped, and segmentation. In patient index *P*_1_, *P*_2_, *P*_4_, and *P*_6_, three operations namely; registration, skull stripping, and segmentation performed successfully with high performance metrics. In *P*_3_, skull stripped was done with low performance of segmentation which detected the tumor and its similar intensity pixels value. In *P*_5_, skull stripped was affected by the limited space between the skull and brain materials, however, its corresponding tumor segmentation was successfully done.

Table 1 shows high performance metrics with a JSC, DSC, FPR, and FNR of range (0.847–0.866), (0.928–0.917), (0.146–0.177), and (0.007–0.003), respectively. Using ground truth of skull-stripped images from three radiologists. This algorithm showed a pronounced value of similarity coefficients (Table 1).

**Table 1:** Summary of the proposed skull-stripping algorithm metrics

Patients (*n* = 120)	Average ± SD			
	JSC	DSC	FPR	FNR
Radiologist_1_	0.86 ± 0.02	0.92 ± 0.01	0.14 ± 0.03	0.007 ± 0.01
Radiologist_2_	0.84 ± 0.03	0.91 ± 0.02	0.17 ± 0.04	0.007 ± 0.01
Radiologist_3_	0.84 ± 0.02	0.91 ± 0.01	0.17 ± 0.03	0.003 ± 0.01

Radiologist_1_, Radiologist_2_, Radiologist_3_ is the first, second, and third radiologist who did manually the skull stripping by 3D Slicer

Based on ground truth of segmented tumor done by three radiologists, Table 2 shows the performance metrics of the tumor segmented with average JSC, DSC, FPR, and FNR of range (0.606–0.676), (0.749–0.803), (0.029–0.11), and (0.258–0.372), respectively. Clearly, the similarity metrics of segmentation is less than the skull stripped. These metrics represent the heterogeneity factor of tumor area where the algorithm of MTS detects the area of pixels greater than the determined threshold.

**Table 2:** Summary of the segmentation metrics

Patients (*n* = 120)	Average ± SD			
	JSC	DSC	FPR	FNR
Radiologist_1_	0.60 ± 0.11	0.74 ± 0.08	0.03 ± 0.04	0.37 ± 0.13
Radiologist_2_	0.64 ± 0.13	0.77 ± 0.10	0.11 ± 0.17	0.29 ± 0.12
Radiologist_3_	0.67 ± 0.09	0.80 ± 0.07	0.09 ± 0.08	0.25 ± 0.13

Radiologist_1_, Radiologist_2_, Radiologist_3_ is the first, second, and third radiologist who segment manually the GBM tumor by 3D Slicer

Moreover, the low intensity of tumor phenotype and the discontinuity of other phenotype area can provide a tumor area segmented like patient *P*_2_. For instance, tumor segmented has a high performance metric like the case of patient *P*_6_ which represents a tumor area with continuity of phenotype area.

A comparative study of skull-stripping algorithm showed a close JSC value of 0.86 and 0.85 considering the proposed algorithm and BET, respectively [9]. Followed by BSE with JSC value of 0.71 (Table 3), [13]. We note that the FPR is common with 0.14 in our work and BET, while BSE model has 0.26 which represents less performance. Moreover, our algorithm showed a best performance value with FNR value 0.007, while BET and BSE models showed a FNR of 0.008 and 0.04, respectively (Table 3). For the specific case of limited subdural space within the image, BSE and BET partially isolate the skull from the brain material which is resolved by the proposed skull-stripping algorithm based on morphology operation.

**Table 3:** Summary of comparative study for skull-stripping measurements

Patients (*n* = 120)	Average ± SD		
	JSC	FPR	FNR
This work	0.86 ± 0.02	0.14 ± 0.03	0.007 ± 0.01
BSE	0.71 ± 0.05	0.26 ± 0.04	0.04 ± 0.04
BET	0.85 ± 0.03	0.14 ± 0.05	0.008 ± 0.02

The proposed method is able to successfully segment the whole brain in all 120 patients images. It has better performance than the two most popular methods in the literature, BET [9, 10], and BSE [13]. However, it outperforms these methods due to its simplicity and speed. We estimate that in group comparison studies of skull stripping, our method can be successfully used. Note that this work is part of a large focus on data analysis of glioblastoma [27–31].

This work showed that our method outperformed the two popular methods for skull stripping, proved to be more sensitive and robust, and most successfully retained brain tissue even within the limited subdural space case. It notes a limitation of the proposed algorithm that is successfully used for axial brain image in 2D, however, BET and BSE works with 3D images.

## Conclusions

In this paper, novel iterative algorithm of skull stripping dedicated to MRI images was proposed. MTS has been developed to detect the tumor area within axial brain image. Preliminary experimental results with 120 patients with tumors confirmed the efficacy of this novel algorithm for automatic skull stripping and brain tumor segmentation in axial images. Moreover, comparison with ground truth of skull stripping and segmented tumor showed that our approach was highly promising for obtaining high performance metrics.

## Abbreviations

BET: Brain extraction tool
BSE: Brain surface extractor
DSC: Dice similarity coefficient
FNR: False negative rate
FPR: False positive rate
FBR: Feature-based registration
FLAIR: Fluid-attenuated inversion recovery
IBR: Intensity-based registration
JSC: Jaccard similarity coefficient
MRI: Magnetic resonance imaging
MIP: Morphological image processing
MTS: Multi-thresholding segmentation
SE: Structuring element
T1-WI: T1-weighted
